# SARS-CoV-2 Establishes a Productive Infection in Hepatoma and Glioblastoma Multiforme Cell Lines

**DOI:** 10.3390/cancers15030632

**Published:** 2023-01-19

**Authors:** Olga A. Smirnova, Olga N. Ivanova, Irina T. Fedyakina, Gaukhar M. Yusubalieva, Vladimir P. Baklaushev, Dmitry V. Yanvarev, Olga I. Kechko, Vladimir A. Mitkevich, Pavel O. Vorobyev, Vyacheslav S. Fedorov, Birke Bartosch, Vladimir T. Valuev-Elliston, Anastasiya L. Lipatova, Alexander V. Ivanov

**Affiliations:** 1 Engelhardt Institute of Molecular Biology, Russian Academy of Sciences, 119991 Moscow, Russia; 2 Gamaleya National Research Centre for Epidemiology and Microbiology of the Ministry of Russia, 123098 Moscow, Russia; 3 Federal Research and Clinical Center of Specialized Medical Care and Medical Technologies of the Federal Medical and Biological Agency of Russia, 115682 Moscow, Russia; 4 Lyon Cancer Research Center/INSERM U1052, 69008 Lyon, France

**Keywords:** SARS-CoV-2, permissiveness, liver cells, hepatoma, glioblastoma

## Abstract

**Simple Summary:**

A novel coronavirus that causes a worldwide pandemic poses a significant threat to patients. The virus can induce not only a cytokine storm and thrombosis but also various extra-respiratory diseases such as liver dysfunction, strong headaches, loss of smell and even psychiatric disorders. However, information on whether SARS-CoV-2 can infect liver or brain tissues is still contradictory. Here, we show that the coronavirus efficiently infects liver cancer cells but does not replicate in non-tumor hepatocyte-like cells. SARS-CoV-2 was also found to infect some glioblastoma cells, which is the most common type of brain tumor. In conclusion, we show that SARS-CoV-2 can infect tumor tissues.

**Abstract:**

Severe acute respiratory syndrome associated coronavirus 2 (SARS-CoV-2) emerged at the end of 2019 and rapidly caused a pandemic that led to the death of >6 million people due to hypercoagulation and cytokine storm. In addition, SARS-CoV-2 triggers a wide array of pathologies, including liver dysfunction and neurological disorders. It remains unclear if these events are due to direct infection of the respective tissues or result from systemic inflammation. Here, we explored the possible infection of hepatic and CNS cell lines by SARS-CoV-2. We show that even moderate expression levels of the angiotensin-converting enzyme 2 (ACE2) are sufficient for productive infection. SARS-CoV-2 infects hepatoma Huh7.5 and HepG2 cells but not non-transformed liver progenitor or hepatocyte/cholangiocyte-like HepaRG cells. However, exposure to the virus causes partial dedifferentiation of HepaRG cells. SARS-CoV-2 can also establish efficient replication in some low-passage, high-grade glioblastoma cell lines. In contrast, embryonal primary astrocytes or neuroblastoma cells did not support replication of the virus. Glioblastoma cell permissiveness is associated with defects in interferon production. Overall, these results suggest that liver dysfunction during COVID-19 is not due to infection of these tissues by SARS-CoV-2. Furthermore, tumors may potentially serve as reservoirs for the virus during infection.

## 1. Introduction

SARS-CoV-2, which emerged in Wuhan at the end of 2019, rapidly spread all over the world and caused a pandemic of coronavirus disease 2019 (COVID-19) [1]. To date, by October 2022, more than 625 million cases of coronavirus infection have been registered, with more than 6.5 million deaths due to infection [2]. SARS-CoV-2 infection is a virus that infects primarily the respiratory track, particularly the lungs. COVID-19 often provokes acute pneumonia, leading to the development of acute respiratory distress syndrome (ARDS) [3]. ARDS is a complex event that involves hyperinflammation, referred to as cytokine storm, and severely activated coagulation leading to thrombosis [4,5]. Both events present a severe threat to patients. The immune response involves mainly the induction of pro-inflammatory cytokines, such as tumor necrosis factor α (TNFα), interleukins (IL) 1β, 6, 8 and others [6], while production of interferons, the antiviral cytokines, remains low (summarized in [7]). Elevated levels of these pro-inflammatory cytokines are also associated with the post-acute sequelae of COVID-19 [8]. The inflammatory response is induced mainly by the Spike (S) and Envelope (E) proteins of the virus, which were found to activate the NF-κB pathway via the Toll-like receptor 2 (TLR2) on a cell surface [9,10]. In addition, in silico analysis suggested that SARS-CoV-2 proteins may use other TLRs on the plasma membrane, including TLR4, to induce inflammatory cytokines [11,12,13]. Again, the major actor is likely to be the S-protein.

As a result, COVID-19 is treated with anti-inflammatory drugs, including glucocorticoids [14] or monoclonal antibodies to cytokines and their receptors [15,16], inhibitors of the respective signaling pathways (i.e., baricitinib) [17] as well as anticoagulatory agents [4]. These drugs along with direct acting antivirals (i.e., paxlovid, molnupiravir and remdesivir) [18,19,20] and introduction of broad vaccination [21] programs significantly improved prognosis of COVID-19 and decreased rates of severe course of the disease. Recently, multiple variants of monoclonal antibodies that protect against severe COVID-19 in patients with various virus variants of concern have been described [22,23,24].

Nevertheless, the virus still poses a significant threat for patients due to the emergence of novel strains, such as omicron variants, that can infect individuals with pre-existing immune responses [25,26] and due to a wide array of pathologies other than inflammation and hyperthrombosis [27,28]. A considerable number of COVID-19 patients continue to suffer after resolution of the infection from “long COVID” symptoms—a wide range of health problems including fatigue, neurological, cardiological, respiratory and other pathologies [29]. Patients with acute infection often demonstrate neurological symptoms [28], signs of mild-to-moderate liver dysfunction [30], altered glucose homeostasis, etc. [31]. This poses the question of whether these pathologies are due to systemic inflammation or whether the virus can infect tissues/cells other than those of the respiratory tract.

Liver dysfunction during COVID-19 has been associated with both hyperinflammatory response and vascular damage and clotting [32]. Indeed, patients with cytokine storms exhibit more pronounced signs of liver damage [33,34] pointing to inflammatory processes as a trigger of the pathology. Levels of proinflammatory cytokines and markers of inflammation correlate with an increase in liver enzymes [34]. Accordingly, among patients who required intensive care treatment, there was a higher incidence of liver dysfunction [34,35]. At the same time, analysis of post-mortem liver samples revealed that levels of markers of inflammation are moderate, while the tissue exhibited signs of multiple thrombosis in intrahepatic vessels [36]. As interleukin 6 was previously shown to induce platelet activation [37], and its level correlates with the elevation of liver enzymes [33], hyperinflammation is likely to promote platelet activation as well as endotheliopathy that drives liver dysfunction [32].

However, it cannot be excluded that SARS-CoV-2 pathology toward the liver is due to direct infection of the organ. Indeed, almost immediately after the identification of SARS-CoV-2, the virus was shown to infect colon cells, in particular enterocytes, which explained the common enteric symptoms of COVID-19 patients [38]. Later, SARS-CoV-2 was reported to infect kidney [39,40], pancreatic cells [41,42] and placenta [43]. Scarce reports exist on the infection of cardiomyocytes, providing another link between infection and cardiological problems [44]. For other organs, the data are vague. Viral RNA has been detected in brain, liver, kidney and blood autopsy samples from deceased COVID-19 patients [45,46]. In the case of the liver, the infectious virus was isolated from this tissue [47], however, this does not confirm infection of hepatocytes. Moreover, SARS-CoV-2 RNA was detected in hepatocytes by in situ hybridization [47]. Nevertheless, infection of primary human hepatocytes and culture of any hepatic cell line has never been shown, with the exception of the human hepatoma Huh7/Huh7.5 cell lines, which are the gold standard for hepatitis C virus research [48,49,50].

Although it was proposed that SARS-CoV-2 can infect cells of the central nervous system (CNS) and several groups infection of astrocytes and, to a lesser extent of neurons [51,52], nothing is known about the permissiveness of brain tumor cells to SARS-CoV-2.

We hypothesized that SARS-CoV-2 could establish productive infection in normal or tumor liver and/or brain cells. Confirmation of this hypothesis would mean that the respective cells/tissues can serve as reservoirs of the virus. Moreover, in this case, the impact of SARS-CoV-2 on these cells/tissues would provide a basis for the development of virus-associated extrapulmonary pathologies. Moreover, the identification of additional types of cells permissive to the infection could point to possible reservoirs of SARS-CoV-2 in the organism and sites for its adaptation to new human tissues. In contrast, if SARS-CoV-2 cannot infect liver and CNS cells, it would mean that liver damage and neurological disorders are indirect and are the consequence of inflammation and hypercoagulation.

Therefore, the goal of this study was to explore if SARS-CoV-2 can infect tumor or non-tumor parenchymal and non-parenchymal liver cells, as well as glioblastoma, neuroblastoma cells or non-tumor astrocytes, to expand knowledge on the tropism of the pathogen.

## 2. Materials and Methods

### 2.1. Reagents

Hydrocortisone, Hoechst 33342, puromycin and DMSO were purchased from Sigma (Darmstadt, Germany). Human recombinant insulin, gelatin, penicillin and streptomycin were provided by Paneco (Moscow, Russia). DMEM, OPTI-MEM and Williams E media were from Gibco (Waltham, MA, USA). Fetal bovine serum (FBS) was from BioSera (Nuaille, France), while Fetal Clone II was purchased from HyClone (Waltham, MA, USA). A High Pure RNA Isolation kit was supplied by Roche (Basel, Switzerland). ABI PRISM^®^ BigDye™ Terminator v. 3.1 kit, Hoechst 33342, Turbofect reagent, RevertAid and Superscript III reverse transcriptases, restriction endonucleases and T4 DNA ligase were from Thermo Fischer Scientific (Waltham, MA, USA). Encyclo DNA polymerase, kits for plasmid DNA purification, pAL-TA vector and oligonucleotides were provided by Evrogen (Moscow, Russia). Matrigel Growth Factor Reduced Basement Membrane Matrix or Matrigel hESC-Qualified Matrix, Collagen I and Laminin were from Corning (Corning, NY, USA). Q5 DNA polymerase was supplied by New England Biolabs (Ipswich, MA, USA).

HepG2 (HB-8065) HEK293T (CRL-3216) A172 (CRL-1620), DBTRG-05MG (CRL-2020), SH-SY5Y (CRL-2266) and A549 (CCL-185) were purchased from American Type Culture Collection (ATCC). Kelly (92110411) and La-N-1 (06041201) were from the European Collection of Authenticated Cell Cultures (ECACC). Huh7.5 cells were a kind gift of Prof. Charles M. Rice (The Rockefeller University, NY, USA) and Apath LLC (St. Louis, MO, USA). LX2 stellate cells were kindly provided by Prof. Scott Friedman (The Icahn School of Medicine at Mount Sinai, NY, USA). Low-passage cultures of primary glioblastoma multiforme (GBM3821, GBM4114, GBM5522 and GBM6138) from surgically removed tumors were obtained at N.N. Burdenko Institute of Neurosurgery (Moscow, Russia) and described previously [53]. Normal embryonic astrocytes have also been previously described [53]. Vero E6 cell lines corresponding to green monkey kidneys were from the Russian National Collection of Cell Cultures at the National Research Center for Epidemiology and Microbiology, named after Honorary Academician N.F. Gamaleya of the Ministry of Health of the Russian Federation (Moscow, Russia). The plasmid pSGR-JFH1 was a gift of Prof. Takaji Wakita (National Institute of Infectious Diseases, Tokyo, Japan), whereas the pLCMV-PL4-puro vector was kindly given by Prof. Peter Chumakov (Engelhardt Institute of Molecular Biology, Moscow, Russia).

### 2.2. Plasmid Construction

Total RNA was purified from the human glioblastoma A172 cell line using a High Pure RNA Isolation kit. cDNA was synthesized from 2 µg of the total RNA using random hexamer oligonucleotide and Superscript III reverse transcriptase, according to the manufacturer’s specifications. ACE2 was amplified using primers 1 and 2 (Table S1) by Q5 DNA polymerase, and the product was cloned into the pAL-TA vector. The ACE2 open reading frame was amplified by oligonucleotides 3 and 4 using the Q5 DNA polymerase, and the product was cloned into the XbaI and EcoRI sites of the pLCMV-PL4-puro vector. 3 U of EcoRI was used to prevent digestion of the inner EcoRI site. The final plasmid was referred to as pLCMV-PL4-ACE2.

HCV IRES was amplified from the pSGR-JFH1 plasmid by primers 5 and 6 and Encyclo DNA polymerase and cloned into EcoRI-XhoI sites of the pLCMV-PL4-ACE2 plasmid yielding the pLCMV-PL4-ACE2-IRES construct.

TMPRSS2 was also amplified from cDNA obtained from A172 cells using oligonucleotides 7 and 8 and Encyclo DNA polymerase. The product was cloned into a pAL-TA vector. Then, TMPRSS2 cDNA was amplified using primers 9 and 10, and the product was cloned into the pLCMV-PL4-ACE2-IRES construct to give the final pLCMV-PL4-ACE2-IRES-TMPRSS2 plasmid.

The sequence of all inserts was confirmed by sequencing using ABI PRISM^®^ BigDye™ Terminator v. 3.1 kit with subsequent product analysis on an Applied Biosystems 3730 DNA Analyzer (Waltham, MA, USA), performed in the DNA sequencing center “Genome” at the Engelhardt Institute of Molecular Biology (Moscow, Russia).

### 2.3. Cell Culture

All glioblastoma and neuroblastoma cell lines, Huh7.5, HepG2 and LX-2 cells, were maintained in DMEM supplemented with 10% FBS, 50 U/mL penicillin and 50 µg/mL streptomycin as described earlier [54,55]. For HepG2 cells, the plates were pre-coated with collagen I, laminin, gelatin, Matrigel Growth Factor Reduced Basement Membrane Matrix or Matrigel hESC-Qualified Matrix. Coating with collagen I was performed by incubation of 6-well plates in a 100-fold diluted collagen solution at 37 °C overnight with subsequent removal of the solution and drying of the cells. Gelatin was applied as a 2% solution in water for 30 min at room temperature. Coating with murine laminin was performed by treating the wells with 10 µg/mL solution 37 °C for 1 h with subsequent washing with PBS. Incubation with Matrigels, diluted 100-fold in cold DMEM, was done at 37 °C for 2 h.

HepaRG cells were cultivated in Williams E medium supplemented with 10% Fetal Clone II, 5 µg/mL insulin, 50 µM hydrocortisone, 50 U/mL penicillin and 50 µg/mL streptomycin (the “Complete” medium). The cells at 95% density were referred to as HepaRG^undiff^. The cells were differentiated as described in [55]. Briefly, they were seeded onto 6-well plates, grown without splitting for 2 weeks after formation of monolayer in the “Complete” medium and then for an additional 2 weeks in the same medium supplemented with DMSO. The resulting culture was referred to as HepaRG^diff^.

### 2.4. Infection Assays

SARS-CoV-2 human coronavirus, passage 3, with infectivity of 10^7.5^ TCID_50_/mL. Strain description: hCoV-19/Russia/Moscow-PMVL-12/2020 (EPI_ISL_572398) GISAD: PMVL-12. Booking reference EPL_ISL_572398. SARS-CoV-2 stock was amplified by infecting confluent Vero E6 cells on 10-cm dishes in DMEM supplemented with 2.5% FBS for 2 h, with gentle shaking every 20 min and subsequent replacement of the medium. The conditioned medium was harvested 96 h post-infection, filtered through a 0.45 µm filter, aliquoted and stored at −80 °C.

The cells were seeded on a 6-well plate 24–48 h prior to infection. Then, the medium was removed, SARS-CoV-2 stock was added in DMEM containing 2.5% FBS at a multiplicity of infection (MOI) of 0.1, and the plate was gently shaken every 20 min. Two hours post-infection, the medium was removed, the cells were washed twice with phosphate buffer saline (PBS), and fresh medium was added (2 mL/well). The conditioned medium and the cells were harvested 4 days post-infection and stored at −20 °C prior the analysis.

### 2.5. Lentivirus Assembly and Cell Transduction

Lentivirus was assembled by transfecting HEK293T cells on a 10-cm dish at 80% density with the resulting plasmid, pLP1, pLP2 and pVSV-G (4, 3, 1.5 and 4 µg, respectively) using Turbofect reagent according to the vendor’s instructions. Briefly, a mixture of the plasmids in 1.9 mL of OPTI-MEM plasmids was vortexed with 24 µL of Turbofect and incubated for 30 min at room temperature prior to the addition of cells in DMEM with 10% FBS. Eighteen hours post-transfection, the medium was replaced with fresh medium. Lentivirus-containing conditioned medium was harvested 48 h post-transfection with subsequent filtering through a 0.45 µm filter, aliquoting and storage at −80 °C.

For lentivirus transduction, Huh7.5 and A549 cells were seeded onto 6-well plates and grown to an 80% monolayer. One hour before transduction, the medium was replaced with OPTI-MEM. For transduction, 200 µL of the respective lentivirus stock was added alongside polybrene to the 10 µg/mL working concentration. Four hours later, the medium was replaced with standard medium. The next day, the cells were split and seeded onto 10 cm dishes. When they reached 90% confluence, puromycin was added to the final concentration of 2 µg/mL for selection of the transduced cells. Four days later, the medium was replaced with standard DMEM containing FBS, penicillin and streptomycin for growth of the polyclonal cell populations.

### 2.6. Reverse Transcription—Real-Time Polymerase Chain Reaction (RT-qPCR)

Total RNA was purified from cells (a well of a 6-well plate per sample) or from culture medium (0.5 mL/sample) using a High Pure RNA Isolation kit. Reverse transcription was carried out from 1 µg RNA using random hexamer primer and RevertAid reverse transcriptase. Real-time PCR was performed as described in [56] using oligonucleotides listed in Table S2. The levels of cellular mRNA or viral RNA in RNA purified from cells were normalized to the levels of β-glucoronidase (GUS) mRNA, whereas the levels of viral RNA in conditioned medium were normalized to its volume.

### 2.7. Immunofluorescence

GBM6138 cells on a 48-well plate were fixed with methanol overnight at −20 °C, washed with PBS (2 × 600 µL). Then, the cells were incubated with 2% solution of bovine serum albumin (BSA) in PBS (500 µL/well) for 1 h at 37 °C with subsequent washing 10 times with PBS (600 µL/well) with gentle shacking. Thereafter, the wells were incubated with 1:50 diluted plasma of a convalescent donor with high levels of antibodies to SARS-CoV-2 proteins [57] in a 2% BSA solution in PBS (200 μL/well) for 1 h at 37 °C. As a negative control, the serum of a donor with no detectable level of antibodies was used. Next, the wells were washed with 600 µL PBS 10 times before the addition of 1:100 diluted FITC-conjugated anti-human IgG (Fc specific) (F9512, Sigma, Darmstadt, Germany) in 1% BSA solution in PBS (200 μL/well). After 45 min incubation at 37 °C, the wells were again washed with 600 µL PBS 10 times with gentle shaking (30 s).

Nuclei were stained by incubation with 5 µg/mL Hoechst 33342 in PBS for 15 min at 37 °C with subsequent washing with 200 μL PBS. The fluorescence was visualized on a ZOE Fluorescent Cell Imager (Bio-Rad, Hercules, CA, USA).

### 2.8. Interferon Production

Sendai virus (Moscow strain) was obtained from the Laboratory of cell proliferation EIMB RAS (Moscow, Russia). UV-inactivated Sendai virus with MOI 5 was added to 50% confluent glioblastoma cells seeded 24 h prior to the experiment. Twenty-four hours later, the cells were harvested, and the total RNA for subsequent RT-qPCR was purified as described above.

### 2.9. Statistical Analysis

All experiments were performed in triplicate at least three times independently. The values presented are the means ± standard deviation (S.D.). The statistical significance of differences between the two groups was assessed by Student’s *t*-test using GraphPad Prism 7 (GraphPad Software Inc., Boston, MA, USA). Multiple comparisons were evaluated by analysis of variance (ANOVA) with Tukey’s post-hoc test.

## 3. Results

### 3.1. Cell Lines Overexpressing ACE2 and TMPRSS2

**Figure 1:**
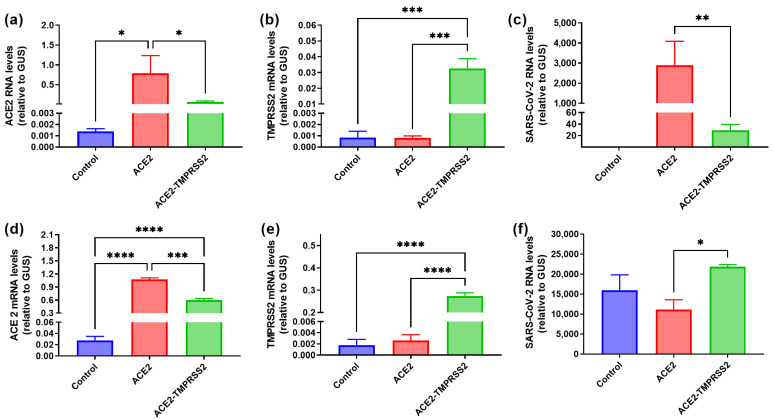
Overexpression of angiotensin-converting enzyme 2 (ACE2) confers the permissiveness of A549 cells to SARS-CoV-2. Naïve A549 (**a**–**c**) or Huh7.5 (**d**–**f**) cells or cells transduced by lentiviruses encoding ACE2 alone or together with TMPRSS2 were infected with SARS-CoV-2 and harvested 4 days post-infection (d.p.i.). Levels of ACE2 (**a**,**d**), TMPRSS2 (**b**,**e**) mRNA or SARS-CoV-2 RNA (**c**,**f**) were assessed by reverse transcription with real-time polymerase chain reaction (RT-qPCR). The levels were normalized to the levels of mRNA of β-glucoronidase (GUS). The values represent the means ± standard deviation (S.D.). * *p* < 0.05, ** *p* < 0.01, *** *p* < 0.001, **** *p* < 0.0001.

Initially, we aimed to screen for lung and liver cell lines permissive to SARS-CoV-2. Since virus entry is dependent on the expression of angiotensin-converting enzyme 2 (ACE2), which is a putative receptor for the virus, and transmembrane protease serine 2 (TMPRSS2) [50], we applied an approach of overexpression of ACE2 either alone or in combination with TMPRSS2. To do this, we constructed mono- and bi-cistronic lentiviruses. In the bi-cistronic lentivirus, ACE2 and TMPRSS2-coding sequences were placed into the same mRNA cassette separated by the hepatitis C virus (HCV) internal ribosome entry site (IRES) with several amino acid residues of the core protein to drive efficient translation of TMPRSS2. Using the respective lentiviruses, two cell lines were transduced: lung adenocarcinoma A549 and human hepatoma Huh7.5. Since the lentiviral vector contained a gene conferring resistance to puromycin, we selected polyclonal populations of these cell lines. Overexpression of both ACE2 and TMPRSS2 was confirmed by reverse transcription and real-time polymerase chain reaction (RT-qPCR). As clearly seen in Figure 1, lentiviral transduction led to an increase in the transcription of the respective genes. Notably, among the control cell lines, Huh7.5 cells expressed ACE2, albeit at a moderate level (Figure 1a,d). Next, we evaluated the permissiveness of these cell lines to SARS-CoV-2. Upon infection, virion production was assessed by measuring genomic viral RNA in the cell supernatants using RT-qPCR. Indeed, overexpression of ACE2 in A549 cells conferred the ability to support SARS-CoV-2 replication (Figure 1c). Naïve Huh7.5 cells that expressed ACE2 at low levels supported efficient replication of the virus, but overexpression of this gene did not potentiate virus replication (Figure 1f). It is worth noting that Huh7.5 cells ensured higher levels of SARS-CoV-2 replication than A549^ACE2^ (Figure 1c,f). Finally, in A549 cells, TMPRSS2 overexpression decreased the permissiveness of cells to the coronavirus, which was associated with a decrease in ACE2 overexpression compared to the monocystronic lentivirus. These results suggest that even moderate expression of the ACE2 receptor is sufficient for cell permissiveness to SARS-CoV-2, and factors other than ACE2 and TMPRSS2 levels modulate levels of the virus replication. In the following experiments as controls, we used A549^ACE2^ cell line as well as Vero E6 cells, which are highly permissive for the infection [58].

### 3.2. SARS-CoV-2 Infects Liver Hepatoma but Not Non-Transformed Hepatocyte-like Cells

The first scientific goal was to investigate the permissiveness of liver cells to SARS-CoV-2. We used naïve human hepatoma Huh7.5 and HepG2 cells, the liver stellate LX2 cell line and the non-transformed liver progenitor HepaRG that can be differentiated into a mixed population of hepatocyte- and cholangiocyte-like cells [55,59]. We then evaluated the permissiveness of HepaRG cells in both undifferentiated and differentiated states.

**Figure 2:**
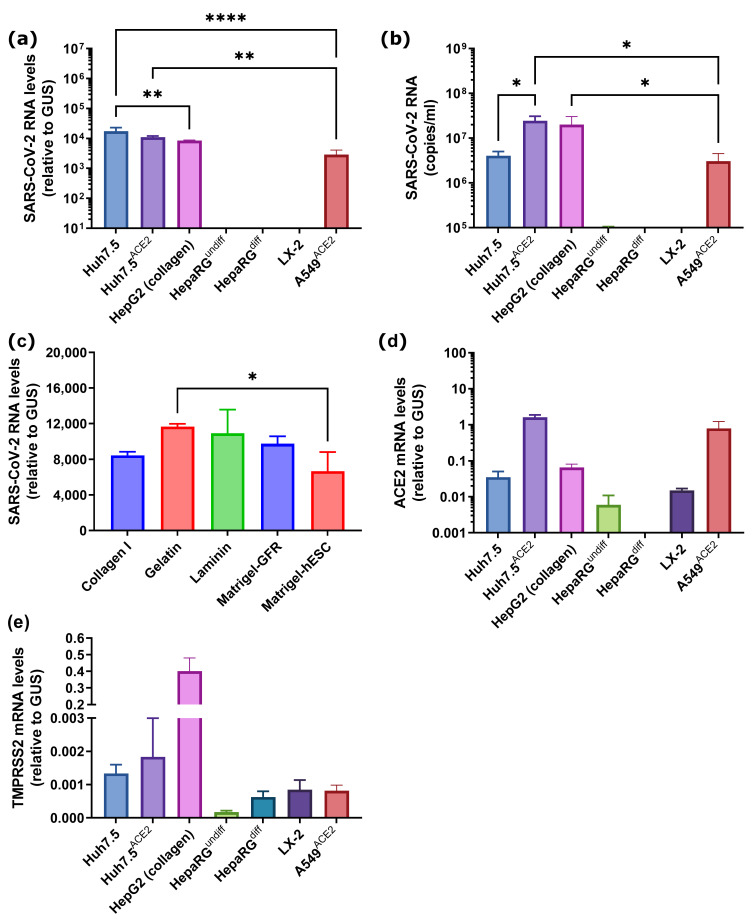
SARS-CoV-2 establishes productive infection in human hepatoma but not in non-transformed hepatocyte-like cells or hepatic stellate cells. Cells were seeded on standard (**a**,**b**) or coated (**c**,**d**) cultured plates (HepG2 cells), incubated with SARS-CoV-2 at a MOI of 0.1 for 2 h, and levels of the virus RNA in cells (**a**,**c**) or conditioned medium (**b**,**d**) were quantified by RT-qPCR 4 days later. (**e**,**f**,) Levels of ACE2 (**e**) or TMPRSS2 (**f**) mRNA in cells presented on panel (**a**). The values are the means ± standard deviation (S.D.). * *p* < 0.05, ** *p* < 0.01, **** *p* < 0.0001.

Moreover, as HepG2 cells, a cell line used for hepatitis B virus (HBV) infection studies, are sometimes maintained on collagen-coated plates for efficient HBV virion production [60], infections were tested not only on plastic with standard coating for adherent cells but also on plates coated with collagen, lamidin, gelatin or two types of Matrigel: Growth Factor Reduced Basement Membrane Matrix (GFR) or Matrigel hESC-Qualified Matrix (hESC). The standard experiment employed incubation of cells with SARS-CoV-2 at MOI 0.1 for 2 h in a medium containing 2.5% fetal bovine serum (FBS) with subsequent removal of medium, two washing steps and incubation of the cells in a fresh media for 4 days. SARS-CoV-2 RNA was analyzed both in the culture supernatant and in the cells. It was found that both hepatoma cell lines (Huh7.5 and HepG2) supported high levels of intracellular SARS-CoV-2 replication and production and secretion of virions into the medium without noticeable signs of the cytopathogenic effect of the virus (Figure 2a–c). The highest intracellular level of viral RNA was observed in naïve Huh7.5 cells, whereas the level of virions in conditioned medium—in Huh7.5^ACE2^ and HepG2 cells. Coating of culture plates for HepG2 cells had no impact on virus replication (Figure 2c). In contrast, neither undifferentiated nor differentiated HepaRG cells showed signs of active infection. This strongly suggests that epithelial liver cells, i.e., hepatocytes and cholangiocytes, are not permissive to the virus. The LX-2 cell line also did not support replication of the virus, thus excluding liver stellate cells from being a possible reservoir of the infection. Permissiveness to infection seems not to be associated with the expression of TMPRSS2, as highly permissive HepG2 and Huh7.5^ACE2^ cell lines demonstrated strong heterogeneity in the expression of this gene.

### 3.3. SARS-CoV-2 Induces Decrease in Expression of Hepatocyte-Specific Markers in HepaRG^diff^ Cells

**Figure 3:**
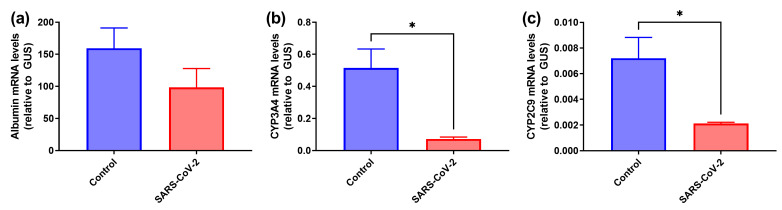
SARS-CoV-2 induces a decrease in the expression of hepatocyte-specific genes. HepaRGdiff cells were infected and incubated in the presence of SARS-CoV-2 for 2 h and expression of albumin (**a**), cytochromes (CYP) 3A4 (**b**) and 2C9 (**c**) was quantified by RT-qPCR 4 d.p.i. The levels were normalized to the levels of GUS mRNA. The values represent the means ± standard deviation (S.D.). * *p* < 0.05.

Next, we evaluated if SARS-CoV-2 can affect the functions of hepatocytes by assessing its impact on gene expression, in particular markers of mature hepatocytes. We measured the levels of transcription of genes encoding albumin and cytochromes (CYP) 3A4 and 2C9 (Figure 3). Interestingly, despite the fact that SARS-CoV-2 does not infect HepaRG cells, it decreases the expression of both cytochromes. In the case of albumin, a tendency close to statistical significance was observed. Therefore, it could be speculated that the coronavirus may induce mild or moderate liver dysfunction via the replication-independent dedifferentiation of hepatocytes.

### 3.4. Several Primary Glioblastoma Cell Lines Support Highly Efficient SARS-CoV-2 Replication

**Figure 4:**
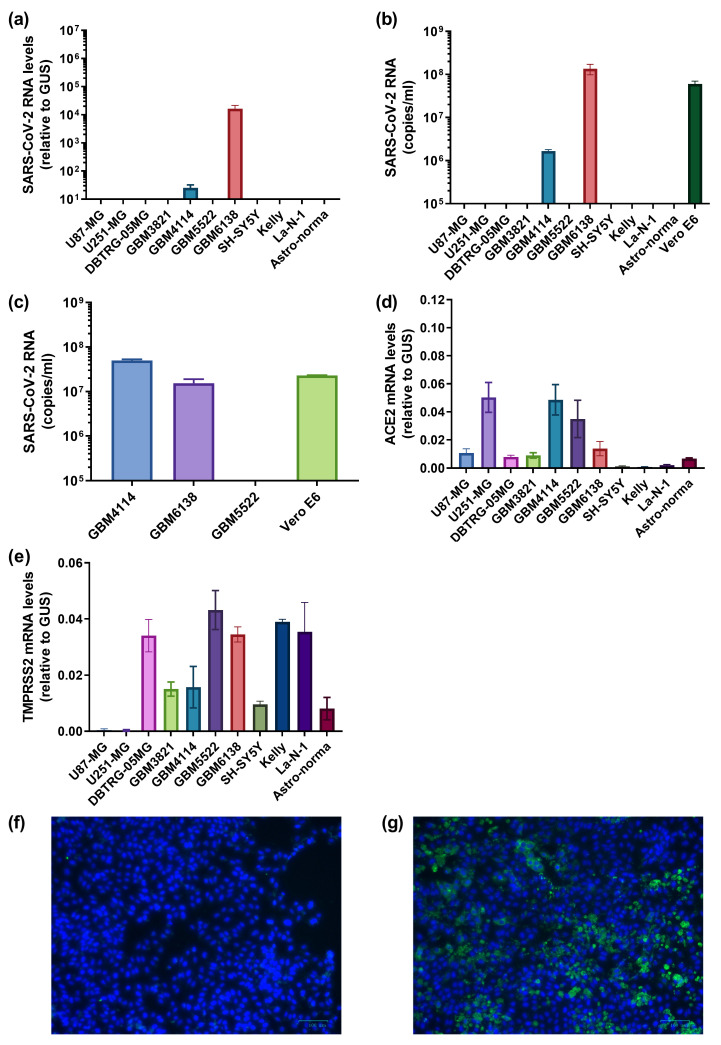
SARS-CoV-2 may establish a productive infection in human high-grade glioblastoma. Glioblastoma U87-MG, U251-MG, DBTRG-05MG, high-grade low-passage glioblastoma (GBM3821, GBM4114, GBM5522 and GBM6138), neuroblastoma (SH-SY5Y, Kelly or La-N-1) or primary embryonic astrocytes were infected with SARS-CoV-2, 2 h later the medium was replaced, and levels of the viral RNA were assessed by RT-qPCR in cells (**a**) or conditioned medium (**b**). (**c**) The conditioned medium from GBM4114, GBM5522 and GBM6138 after infection was used to treat Vero E6 cells, and SARS-CoV-2 RNA in media was monitored by RT-qPCR. (**d**,**e**) Levels of ACE2 and TMPRSS2 mRNAs were measured in glioblastoma and neuroblastoma cell lines. The values are the means ± standard deviation (S.D.). (**f**,**g**) Immunostaining of the GBM6138 cell line infected with SARS-CoV-2 using non-immune serum (**f**) and immune (**g**) serum.

Our next step was to evaluate if SARS-CoV-2 can infect cells of the CNS. As such, we examined Astro-norma, an embryonic astrocyte cell line, as well as a series of neuroblastoma (SH-SY5Y, Kelly and La-N-1) and glioblastoma cell lines (U87-MG, U251-MG and DBTRG-05MG). The list also included four low-passage cells established from high-grade glioblastoma resections (GBM3821, GBM4114, GBM5522 and GMB6138). Initially, these cells were incubated with SARS-CoV-2 for 2 h, similar to VeroE6 or A549 cells, and later washed with fresh medium to remove virions, and replication of the virus was assessed 4 days later by quantifying its RNA in cells and media (Figure 4a,b). Neither primary embryonic astrocytes nor neuroblastoma cells revealed any signs of SARS-CoV-2 replication. Strikingly, viral RNA was detected in two variants of low-passage primary glioblastoma cells, namely GBM4114 and GBM6138. The replication in the latter was even higher than in classical highly permissive Vero E6 cells. Additionally, SARS-CoV-2 did not exhibit a cytotoxic effect forwarding these cell lines. In order to show that these cells produced not just some virus-like particles or defective virions but infectious virions, the conditioned medium was used to infect Vero E6 cells. Indeed, the medium from these two cell lines but not from GBM5522, established classical infection (Figure 4c), showing that SARS-CoV-2 can productively infect some glioblastoma cells. Productive infection of GBM6138 was also verified by immunostaining the cells infected with SARS-CoV-2 4 dpi using immune serum (Figure 4f,g). Finally, we quantified the levels of ACE2 and TMPRSS2 mRNA in all of these cell variants (Figure 4d,e). It was found that the permissive GBM4114 and GBM6138 cell lines exhibited relatively higher levels of transcription of ACE2 compared to the non-permissive cell lines, with the exceptions of U251-MG and GBM5522. This indicates that other factors also control the permissiveness of cells to the coronavirus.

### 3.5. The Highly Permissive Glioblastoma GBM6138 Cell Line Has Defects in Type I Interferon Production

**Figure 5:**
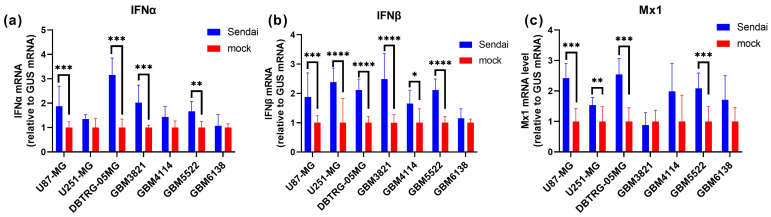
The hyperpermissive GBM6138 cell line has an impaired type I interferon system. Glioblastoma U87-MG, U251-MG, DBTRG-05MG, GBM3821, GBM4114, GBM5522 and GBM6138 were infected with Sendai virus, preinactivated with ultraviolet, and 24 hpi expression of interferons α (**a**) and β (**b**) as well as Mx1 (**c**) gene was assessed by RT-qPCR in cells. The values are the means ± standard deviation (S.D.). * *p* < 0.05, ** *p* < 0.01, *** *p* < 0.001, **** *p* < 0.0001.

We also assessed the interferon system status in glioblastoma cells, as type I interferons are the key mediators of antiviral immunity [61]. We measured the expression of interferons α and β (IFNα, IFNβ) as well as of MX Dynamin Like GTPase 1 (Mx1), one of the interferon-stimulatory genes (ISGs). UV-inactivated Sendai virus was used at high MOI as inducer of IFN production, [62]. Levels of transcription of the abovementioned genes, depicted in Figure 5, clearly show that among various glioblastoma cell lines, GMB6138 is incapable of producing type I interferons. In line with this, this cell line also showed no signs of upregulation of Mx1 gene transcription in response to the Sendai virus. Therefore, GMB4114, which was moderately permissive to SARS-CoV-2, exhibited a low, although statistically significant, increase in IFNβ gene transcription. The non-permissive cell lines were capable of IFNβ production in response to treatment with the inactivated Sendai virus.

## 4. Discussion

COVID-19 patients, adult, adolescent and pediatric, often exhibit signs of liver dysfunction, such as elevated levels of liver enzymes in blood (ALT, AST, GGT), hypoalbuminemia and abdominal pain [63,64,65]. Moreover, the risk of liver pathology is increased in cohorts of patients with severe COVID-19 and an increase in ALT/AST/GGT is an independent predictor of adverse effects during coronavirus disease and concomitant hospital admission [64,66]. Cases of liver failure in patients with no previous history of liver pathology have also been reported [67]. In patients with pre-existing liver pathologies (such as cirrhosis), the incidence of liver decompensation significantly increases and the death rate more than doubles [68]. Opposite reports, i.e., on the absence of correlation between liver dysfunction and mortality rates of COVID-19 patients, also exist [69]. Therefore, investigation of the mechanisms of SARS-CoV-2 pathogenesis that impact the liver merits study.

The liver is comprised of parenchymal and nonparenchymal cells. Parenchymal cells are hepatocytes and cholangiocytes, whereas non-parenchymal cells include hepatic stellate cells (HSCs) that are responsible for fibrogenesis, Kupfer cells (liver-residing macrophages) and sinusoidal cells (endothelial) [70]. Previously, Puelles et al. detected SARS-CoV-2 RNA in liver post-mortem samples [46]. This study was corroborated by Wanner et al., who detected viral RNA specifically in hepatocytes and isolated the infectious virus from liver tissues, which suggested active replication in this organ [47]. Importantly, hepatocytes in the context of the liver were shown to express the ACE2 receptor. However, it cannot be completely excluded that SARS-CoV-2 does not infect hepatocytes but is merely absorbed by them with no subsequent replication, whereas the infectious particles may have been purified from the sinusoids. Here, we showed that hepatocyte- and cholangiocyte-like cells are non-permissive to SARS-CoV-2. Thus, liver dysfunction that can occur during COVID-19 is likely to originate from systemic inflammation. Nevertheless, as observed in this study, the decrease in the expression of hepatocyte-specific genes in differentiated HepaRG cells may also point to the virus as a direct trigger of hepatocyte dysfunction through some yet undiscovered mechanism.

SARS-CoV-2 infection, at least in the case of pre-omicron variants, was associated with neurological symptoms such as attention disorders, anosmia, ageusia, ataxia, headaches and dizziness, seizures as well as confusion and in some cases even psychiatric disorders [71,72]. High-resolution magnetic resonance imaging (MRI) revealed a reduction in cortical thickness in the left hemisphere of the brain of even patients with mild COVID-19 [52]. Therefore, it was logical to explore if the virus can infect various cells of the CNS. SARS-CoV-2 infects the choroid plexus epithelium, leading to its dysfunction and disruption of the blood-brain barrier [73,74,75]. Several groups have shown that SARS-CoV-2 can establish productive infection in cortical astrocytes and in neurons [51,52,76,77], neural progenitor cells [77], glial cells of the cortex [74] and brain organoids [76,77]. Beckman et al. evidenced replication of the virus in neurons of macaques with subsequent induction of neuroinflammation [78]. Nevertheless, several studies have concluded that SARS-CoV-2 does not infect neurons [51,73,74,75]. Wang et al. also did not remark efficient replication in astrocytes [76]. Thus, more studies are needed to resolve these discrepancies.

Our study contributes to the field by showing that SARS-CoV-2 can infect brain tumors, specifically glioblastoma multiforme, at least in some cases. Nevertheless, in these cases, levels of replication are high and even exceed levels observed in a highly permissive Vero E6 cell line, considered as a gold standard in the field. Previously, SARS-CoV-2 RNA and proteins were detected in tumor samples from a convalescent patient with GBM [79]. Bielarz et al. reported that the virus can infect glioblastoma U87-MG and U373-MG cells and neuroblastoma SH-SY5Y and SK-N-BE(2) neuroblastoma cells differentiated with retinoic acid, albeit at a very high MOI [80]. These data do not contradict our findings, as we used much lower MOIs. Indeed, using similarly low MOIs, the above-mentioned authors also did not detect productive coronavirus replication. Moreover, more recently, Vanhulle et al. showed that the U87-MG cell line is non-permissive to SARS-CoV-2, unless the ACE2 receptor is overexpressed [81].

The ability of SARS-CoV-2 to infect hepatoma and some variants of glioblastoma and its incapacity to infect non-transformed liver or neuroblastoma cells is at least partially associated with the expression of the ACE2 receptor. In our hands, the level of ACE2 expression did not correlate with the efficacy of virus replication, pending that the cells expressed this gene at least at a moderate level. Initially, this was suggested by the non-permissiveness of A549 cells and the permissiveness of Huh7.5 cells in which the level of ACE2 mRNA was 0.1% and 2% of a housekeeping GUS mRNA (Figure 1). Moreover, an increase of its level in Huh7.5 cells to 90% (in the case of overexpression) did not affect SARS-CoV-2 replication. In the other permissive liver cell line, HepG2 cells, the ACE2 mRNA level was approximately 6% of GUS mRNA, while in the non-permissive HepaRG and LX-2 cells—at 0.1% and 1%, respectively (Figure 2). Among the glioblastoma/neuroblastoma cell lines, the permissive MGB4114 and MGB6138 were also characterized by increased expression of ACE2 (4.9 and 1.4% of GUS mRNA, respectively), compared to low expression for the majority of non-permissive cell lines (0.09–1% in general). However, there were two exceptions: U251-MG and MGB5522 glioblastoma cell lines (5 and 3.5%). Therefore, the expression of ACE2 is not the sole factor in cell permissiveness to SARS-CoV-2. No correlation was observed between viral replication and the expression of TMPRSS2, as is clearly seen in the dataset for glioblastoma/neuroblastoma cells. These data align with the findings for astrocyte infection, which was shown to depend on CD147 and dipeptidyl protease 4 (DPP4/CD26) co-receptors [51]. Both these proteins are expressed in a wide array of tissues, including the liver and brain and are required for their pleiotropic functions [82,83]. In CNS cells, especially those of tumor origin, SARS-CoV-2 infection was also shown to depend on the expression of cathepsins B and L [28]. Another co-receptor for the virus is Neuropilin 1 (NRP1) [52]. However, in our study, the expression of all these co-receptors has not been analyzed. In addition, we did not assess differences in the permissiveness of cells to various variants of concern for SARS-CoV-2. In contrast, we showed that the highly permissive glioblastoma GBM6138 cell line was deficient in type I interferon production. Since recombinant interferon demonstrates anti-SARS-CoV-2 activity [84], and the virus itself can enhance IFN production [62], we can draw parallels with the highly permissive HCV Huh7.5 cell line that is also incapable of IFN responses [85,86]. Moreover, since neoplastic transformation is often accompanied by downregulation or even inactivation of the interferon innate response [87], this suggests that SARS-CoV-2 may infect tumor tissues.

Altogether, our study shows that SARS-CoV-2 can infect liver and brain tumor cells, thus establishing a reservoir for the infection. It has several clinical implications. First, given that SARS-CoV-2 entry does not necessarily lead to cell death [50,80], the infection may cause prolonged virus shedding. Second, in these cases, the virus may also act as an oncolytic agent, leading to tumor reduction. Indeed, such cases for hematological patients have already been described in the literature [88,89,90]. Though we by no means consider the usage of viable SARS-CoV-2 for virotherapy rational, as the infection exhibits high mortality in cancer patients [91], we cannot exclude the possibility that certain proteins of the virus can be utilized for construction of recombinant oncolytic viruses. Such studies have already been carried out [92,93]. Third, infection of various tissues with the establishment of a prolonged reservoir can result in adaptation of the virus to tissues by introduction of mutations in its genome [94]. This can lead to the appearance of new variants with different immunogenicity, infectivity and pathogenesis. Bukh’s group already showed that passaging SARS-CoV-2 in human cell lines is accompanied by the introduction of adaptive mutations that affect virus titers and increase its cytopathic effect [95]. In addition, such viruses may escape from naturally occurring antibodies or therapeutic neutralizing antibodies that are widely developed for treating immunocompromised patients [22,23].

Finally, our study has a very important limitation: our findings were obtained from in vitro models. Therefore, the finding that SARS-CoV-2 can infect hepatocarcinoma and at least some variants of glioblastoma cancer cells has to be verified by analysis of viral RNA in liver/brain tumor tissues from cancer patients with COVID-19. Second, prolonged virus shedding has to be re-evaluated in these patients, as it can also support the conclusion about the existence of the SARS-CoV-2 reservoir in tumors.

## 5. Conclusions

In conclusion, in the present study, we show that SARS-CoV-2 can infect hepatocarcinoma and some glioblastoma cell lines. Therefore, these cell lines can be used to study the molecular biology and pathogenesis of beta-coronaviruses, including SARS-CoV-2. These data point to the investigation of SARS-CoV-2 replication in cancer patients as a future direction for the study.

## Data Availability

The data presented in this study are available in this article.

## Supplementary Materials

The following supporting information can be downloaded at: https://www.mdpi.com/article/10.3390/cancers15030632/s1, Table S1: Oligonucleotides used for plasmid construction; Table S2: Oligonucleotides used in real-time PCR.
