# Supplementary material for: SARS-CoV-2 Establishes a Productive Infection in Hepatoma and Glioblastoma Multiforme Cell Lines
Source: Cancers (Basel). 2023 Jan 19;15(3):632. doi: 10.3390/cancers15030632 (PMC9913867; doi:10.3390/cancers15030632)
Supplement: Supplementary file 1 [file cancers-15-00632-s001.zip › cancers-2075306-supplementary.pdf]

SUPPLEMENTARY DATA

SARS-CoV-2 establishes a productive infection in hepatoma and glioblastoma multiforme cell lines

Olga A. Smirnova [1,#], Olga N. Ivanova [1,#], Irina T. Fedyakina [2], Gaukhar M. Yusubalieva [1,3], Vladimir P. Baklaushev [1,3], Dmitry V. Yanvarev [1], Olga I. Kechko [1], Vladimir A. Mitkevich [1], Pavel O Vorobiev [1], Vyacheslav S. Fedorov [1], Birke Bartosch [4], Vladimir T. Valuev-Elliston [1], Anastasiya L. Lipatova [1] and Alexander V. Ivanov [1,*]

[1] Engelhardt institute of molecular biology, Russian academy of sciences, Moscow, Russia
[2] Gamaleya National Research Centre for Epidemiology and Microbiology of the Ministry of Russia, Moscow, Russia
[3] Federal Research and Clinical Center of Specialized Medical Care and Medical Technologies FMBA of Russia, Moscow, Russia
[4] Lyon Cancer Research Center / INSERM U2052, Lyon, France
* Correspondence: aivanov@yandex.ru
[#] These authors contributed equally

Table S1. Oligonucleotides used for plasmid construction.

Primer ID	Primer name	Sequence (5′-3′)
1	ACE2-nst-F	AGTCTAGGGAAAGTCATTCAGTGG
2	ACE2-nst-R	TCTCTCCTTGGCCATGTTGTC
3	ACE2-F	ATTTCTAGAATGTCAAGCTCTTCCTGGCT
4	ACE2-R	ATTGAATTCCTAAAAGGAGGTCTGAACATCATCAG
5	IRES-F	ATTGAATTCTAGGTTTAAACCCTCTCCCTCCC
6	IRES-R	ATTCTCGAGAGCCATGGTATCATCGTGTTTTTCAAAG
7	TMPRSS2-nst-F	CTGGGGAGGGGAACCTGG
8	TMPRSS2-nst-r	ACAGAATGGCAGAGAGTGCC
9	TMPRSS2-F	ATTCTCGAGATGCCCCCTGCCCC
10	TMPRSS2-R	ATTGTCGACTTAGCCGTCTGCCCTCATTTG

[1] Tables may have a footer.

Table S2. Oligonucleotides used in real-time PCR.

Gene	Orientation	Sequence (5′-3′)
SARS-CoV-2	Sense	CACATTGGCACCCGCAATC
	Antisense	GAGGAACGAGAAGAGGCTTG
ACE2	Sense	CGAAGCCGAAGACCTGTTCTA
	Antisense	GGGCAAGTGTGGACTGTTC
TMPRSS2	Sense	CAAGTGCTCCAACTCTGGGAT
	Antisense	AACACACCGATTCTCGTCCTC
Albumin	Sense	TGCTTGAATGTGCTGATGACAGGG
	Antisense	AAGGCAAGTCAGCAGGCATCTCATC
CYP3A4	Sense	CCTTACACATACACACCCTTTGGAAGT
	Antisense	AGCTCAATGCATGTACAGAATCCCCGGTTA
CYP2C9	Sense	CCTCTGGGGCATTATCCATC
	Antisense	ATATTTGCACAGTGAAACATAGGA
IFNα	Sense	GTGAGGAAATACTTCCAAAGAATCAC
	Antisense	TCTCATGATTTCTGCTCTGACAA
IFNβ	Sense	GCCGCATTGACCATGTATGAGA
	Antisense	GAGATCTTCAGTTTCGGAGGTAAC
Mx1	Sense	GGTGGTCCCCAGTAATGTGG
	Antisense	CGTCAAGATTCCGATGGTCCT
GUS	Sense	CGTGGTTGGAGAGCTCATTTGGAA
	Antisense	ATTCCCCAGCACTCTCGTCGGT
